# Estimating Microtubule Distributions from 2D Immunofluorescence Microscopy Images Reveals Differences among Human Cultured Cell Lines

**DOI:** 10.1371/journal.pone.0050292

**Published:** 2012-11-28

**Authors:** Jieyue Li, Aabid Shariff, Mikaela Wiking, Emma Lundberg, Gustavo K. Rohde, Robert F. Murphy

**Affiliations:** 1 Center for Bioimage Informatics, Carnegie Mellon University, Pittsburgh, Pennsylvania, United States of America; 2 Lane Center for Computational Biology, Carnegie Mellon University, Pittsburgh, Pennsylvania, United States of America; 3 Department of Biomedical Engineering, Carnegie Mellon University, Pittsburgh, Pennsylvania, United States of America; 4 Department of Electrical and Computer Engineering, Carnegie Mellon University, Pittsburgh, Pennsylvania, United States of America; 5 Department of Biological Sciences and Department of Machine Learning, Carnegie Mellon University, Pittsburgh, Pennsylvania, United States of America; 6 Faculty of Biology and Freiburg Institute for Advanced Studies, Albert Ludwig University of Freiburg, Freiburg, Germany; 7 Science for Life Laboratory, Department of Biotechnology, Royal Institute of Technology, Solna, Sweden; Glasgow University, United Kingdom

## Abstract

Microtubules are filamentous structures that are involved in several important cellular processes, including cell division, cellular structure and mechanics, and intracellular transportation. Little is known about potential differences in microtubule distributions within and across cell lines. Here we describe a method to estimate information pertaining to 3D microtubule distributions from 2D fluorescence images. Our method allows for quantitative comparisons of microtubule distribution parameters (number of microtubules, mean length) between different cell lines. Among eleven cell lines compared, some showed differences that could be accounted for by differences in the total amount of tubulin per cell while others showed statistically significant differences in the balance between number and length of microtubules. We also observed that some cell lines that visually appear different in their microtubule distributions are quite similar when the model parameters are considered. The method is expected to be generally useful for comparing microtubule distributions between cell lines and for a given cell line after various perturbations. The results are also expected to enable analysis of the differences in gene expression underlying the observed differences in microtubule distributions among cell types.

## Introduction

Microtubules play an indispensable role in subcellular processes such as cell movement, cell division and intracellular transportation. In turn, these processes are known to play a role in other biological phenomena such as wound healing, and cancer metastasis. Extracting information about the organization of microtubules in different cell lines could potentially shed light on the roles of microtubule associated proteins in that organization. While limited information is available about variation in microtubule distributions [1], [2], information on those distributions in intact cells for different cell lines has not been readily available. Most microtubule studies have focused on dynamics and interactions with drugs and microtubule associated proteins [3]–[6]. We believe that the ability to obtain reliable estimates of the overall organization of microtubules in whole cells could allow quantification of their dependency on different pertubagens, drugs, mechanical stimuli, etc.

Electron microscopy can be used to trace microtubules, but the specimen preparation for imaging does not allow for intact cells to be imaged. Fluorescence microscopy can be used to image intact cells, but microtubules typically overlap and are often densely packed inside cells. It is very difficult, if not impossible, to manually trace each individual microtubule in a confocal or wide-field fluorescence microscopy image in order to obtain accurate estimates of microtubule distribution parameters. Hence previous work comparing cell lines has often focused on the tips of microtubules where tracing is possible, or the comparison has been only qualitative [7].

**Figure 1:**
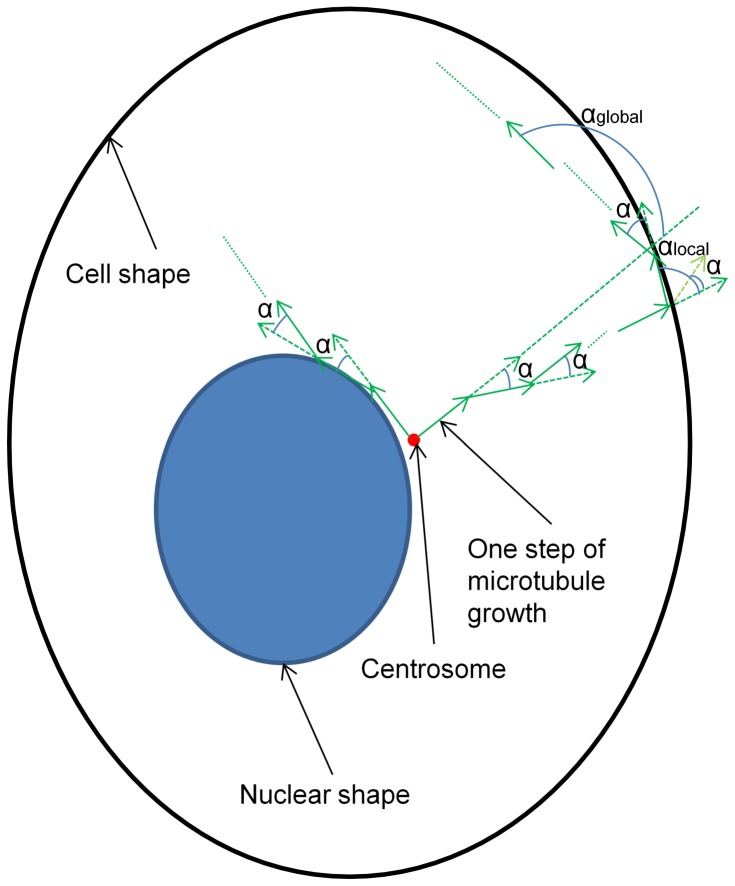
Growth model for generating microtubules dependent on cell and nuclear shapes. Each microtubule starts from the centrosome, and randomly grows to the second point on the lateral surface of a cone whose aperture is 2α. Then the microtubule grows the same way until it hits the cell or nuclear shape boundary and is not able to step further within the cytosolic area. At this time, we relax the collinearity requirement but still confine the next direction under the local constraint α_local_. Moreover, we also keep on checking a consecutive multiple (30) steps, and require that there are less than or equal to 3 pairwise vector angles that are larger than the global constraint α_global_. Beginning with an empty (black) cytosolic area (shaped by cell and nuclear boundary), we add one to the intensity of the pixel which a microtubule crosses. In this paper, we used every step of growth to be 0.2 microns (1 pixel). For the two constraints on the collinearity which controls the curvature of each microtubule and the local and global rebounding issues, we used α_local_ to be 63.9 degrees and α_global_ to be 120 degrees. The figure only illustrates the procedure of growth in 2D for better visualization but can be easily imagined to extend to 3D.

We therefore previously developed an indirect method for estimating natural, interpretable and quantitative parameters such as the number and the mean length of microtubules from 3D fluorescence microscopy images of microtubules [8], [9]. These parameters are important because they represent basic biophysical characteristics of tubulin polymerization. The basis of the method is to use a generative model of microtubule patterns (**Figure 1**) to synthesize 3D images for many values of the model parameters, and then to pick the image that best matches the given real image (and thus to estimate the parameters that could have produced it). Our original method utilized 3D images, but 3D images of intact whole cells are much less commonly available than 2D images. We therefore describe here a method of estimating 3D microtubule model parameters from 2D image fluorescence microscopy images of tubulin. We test our approach on the 3D images of HeLa cells previously used to develop the model, and then use it to compare microtubule distributions in different cell lines.

**Figure 2:**
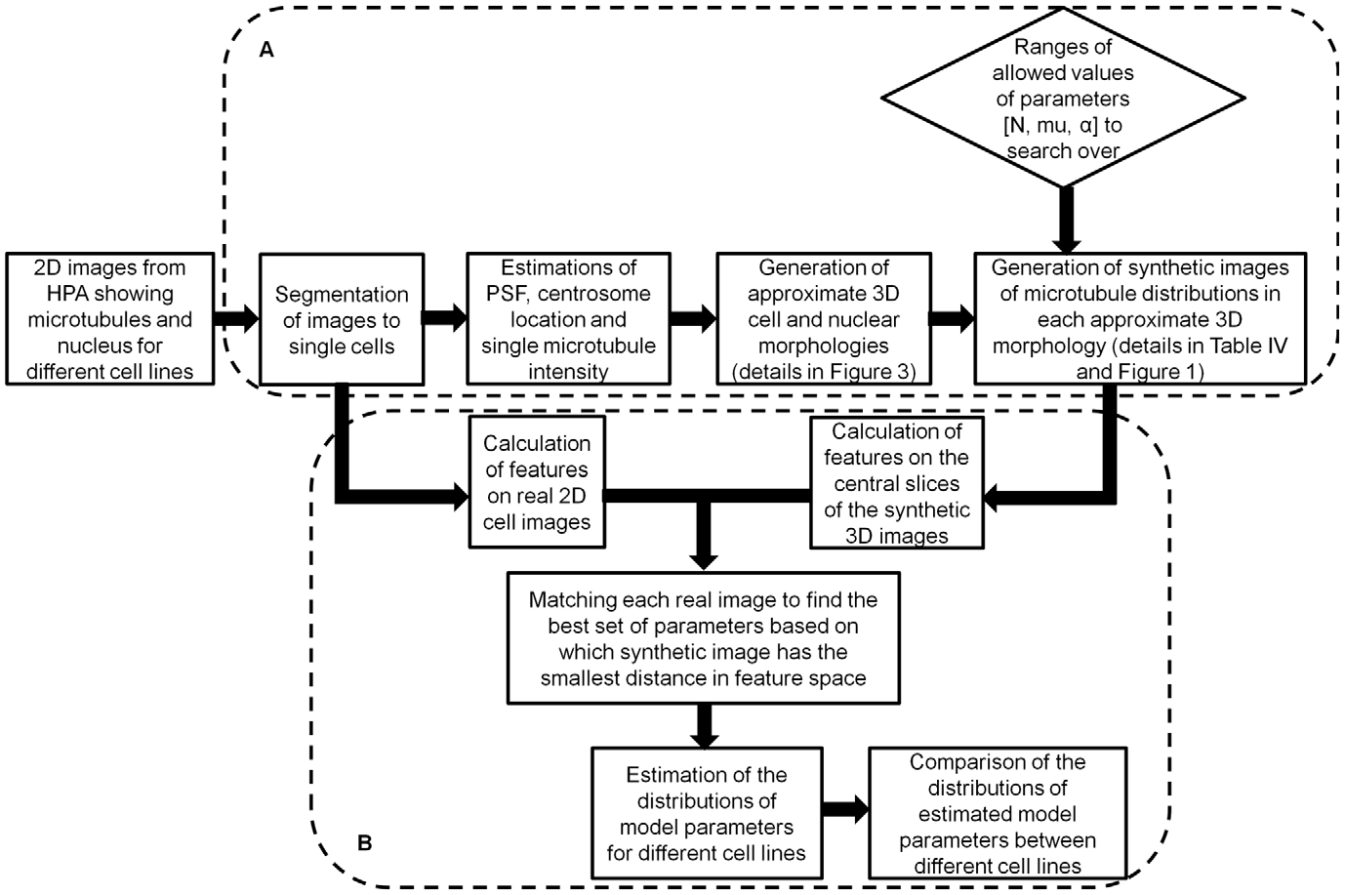
An overview of the framework introduced in this paper. The framework contains two sub-systems, one for generating 3D synthetic images of distributions of microtubules (A), and one for estimating and comparing the model parameters of distribution of microtubules from real 2D images of eleven cell lines (B).

**Figure 2** provides an overview of the framework introduced in this paper. There are two sub-systems. One is for generating synthetic images of microtubules, and the other is for estimating the microtubule model parameters for real images through comparison with the synthetic images. We first obtained 2D fluorescence microscopy images for eleven cell lines. Each image contains two channels, one for microtubule staining and the other for nuclear staining. The images are segmented to find individual cell and nuclear boundaries. For each cell, we estimate a Point Spread Function (PSF), centrosome location and single microtubule intensity. On the basis of the segmented 2D cell and nuclear shapes, approximate 3D cell and nuclear morphologies are generated. Given the model (**Figure 1**) and ranges of allowed values of its parameters (number of microtubules (N), mean of the length distribution (mu) and collinearity (α)), synthetic images of microtubule distributions are generated for each 3D morphology for each combination of allowed parameter values. Each raw synthetic image is then convolved with the estimated PSF and multiplied with the estimated single microtubule intensity to make it comparable to the real image. Numerical features are then calculated on every real cell image and the synthetic images for it. The matching method then selects one set of parameters for which the synthetic image is the closest to the real image in the feature space.

Using this indirect method, we estimate the model parameters for 2D images from eleven human cell lines, and analyze the resulting parameters.

## Results

### 3D Cell and Nuclear Shape Generation from a 2D Slice of Microtubule Channel and Nucleus Channel

**Figure 3:**
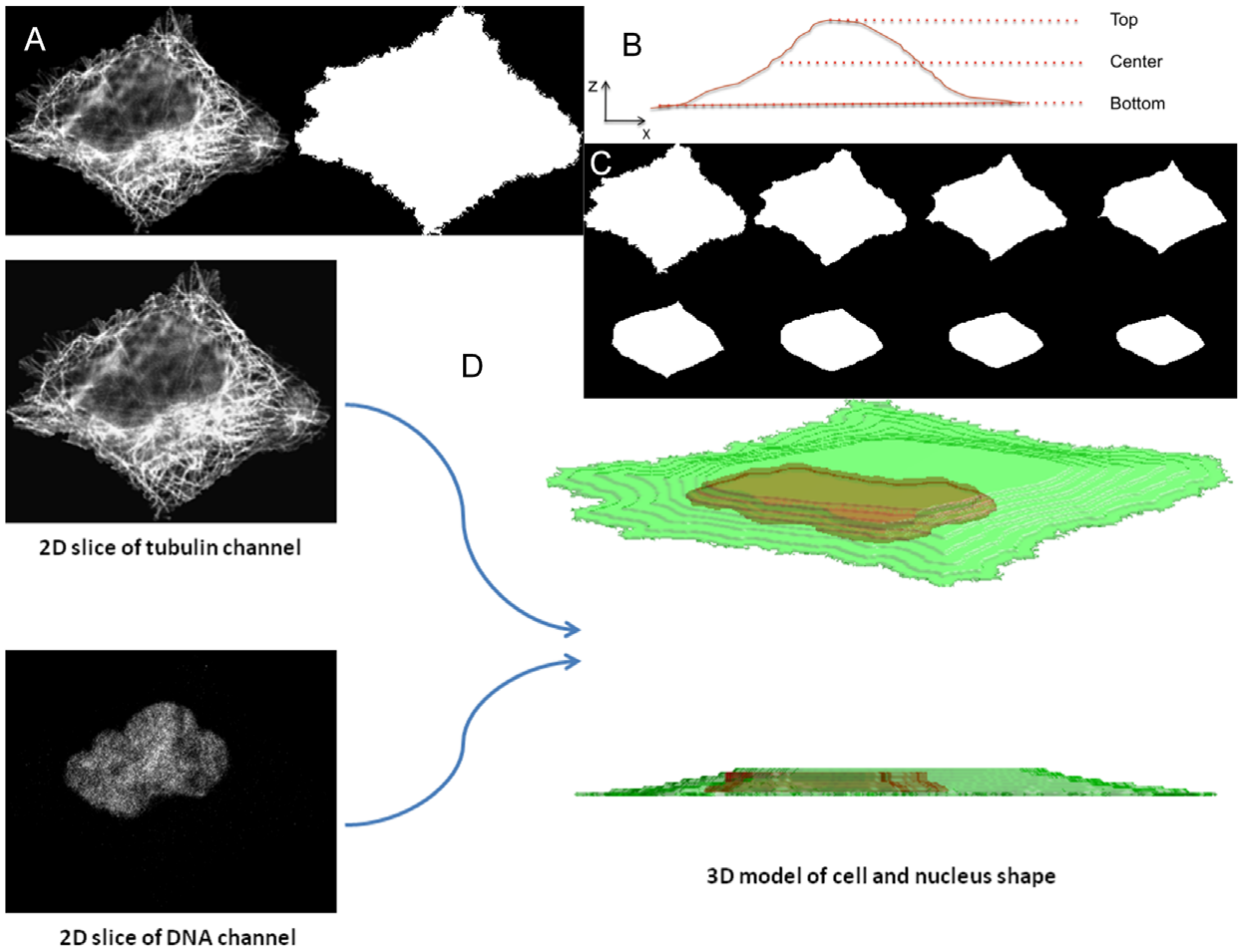
Generation of 3D cell geometry (cell shape and nuclear shape) from real 2D slices of the microtubule and nucleus channels. (**A**) Example of a real 2D cell image (tubulin channel) and its approximate bottom shape. (B) Cartoon of an X-Z projection of a cell on a substrate. (C) Example of a generated 3D cell shape containing 8 stacks (1.6 microns). (D) Illustration of inputs and outputs for the procedure.

In our earlier work, we described an indirect approach to estimate parameters of a generative model of microtubules that was conditioned on the shape of the cell and the nucleus [8]. These shapes were estimated from a 3D confocal stack of images of a total protein stain and a DNA stain respectively. Since the images we analyze in this paper are only 2D slices, we developed an approach to estimate an approximate 3D shape of a cell and nucleus from a 2D slice (purely for the purpose of being able to generate a synthetic microtubule distribution). The location of the centrosome was also estimated (see Methods). **Figure 3** shows an example of microtubule and nucleus images and the resulting approximate 3D cell and nucleus shape models (see details in the section of “3D cell and nuclear morphology generation” in Methods). We also describe a method to detect the 3D coordinate of the centrosome from the microtubule image using a two step approach (see Methods). These models and centrosome location were then used to generate microtubules in the cytosolic space.

### Recovering 3D Microtubule Generative Model Parameters from 2D Images: comparisons with real 3D estimates

To test the accuracy of estimating microtubule parameters from 2D images, we applied our new 2D method (see Methods) using the central slice (at half height of the cell) of 3D HeLa cell images and compared the estimated parameters with those from the 3D method. The half height was chosen as the preferred slice because the 2D images used later were also acquired at half the height of the cell. We computed the mean absolute percentage error (MAPE) in each of the parameters estimated from the 2D images assuming that the estimated parameters from the 3D method were correct. Results are shown in **Table 1** for 42 cells. From the table, we can see that the estimates of the number of microtubules and collinearity from a single 2D slice are reasonably close to those from the entire 3D image. However, the MAPE for the mean length appears to be somewhat larger. We will aim to reduce this discrepancy in future work. However, we note that most cells were estimated to have mean length of 10 or 15 microns (see the section of library generation in Methods) using the 3D method on the original 3D images. Therefore a small deviation in the estimates of 5 microns (the increment of the range of allowed values of mean length) would cause a MAPE of 50 or 33. The table also shows a comparison of the true cell heights and the estimated ones, with the results showing that they are reasonably close.

**Table 1:** Comparisons of estimated parameters of distribution of microtubules between original 3D HeLa images and their 2D central slices.

Number of microtubules	Mean of length distribution	Collinearity (cos*α*)	Cell Height
23.9±19.7	43.1±23.9	1.96±2.72	21.4±13.1

The values in the second row are MAPEs of the recoveries of parameters from the 2D slices, assuming that the parameter estimates from the 3D images are correct.

### Recovering 3D Microtubule Generative Model Parameters from 2D Images: simulated experiments

We estimated how well our recovery method can perform using simulated images for which the correct parameters were known. For one cell geometry (cell shape and nucleus shape), a library of 3D synthetic images was generated with predefined parameters as a validation bed; then 5 other testing libraries were generated using different random seeds. The predefined parameters for the validation bed were estimated from each testing library separately. The resulting errors are shown in **Table 2** and indicate that the system accurately recovers model parameters from 2D slices of synthetic images.

**Table 2:** Estimated accuracies of recovery of model parameters from synthetic 2D images in the simulation experiment.

Library	Number of microtubules	Mean of length distribution	Collinearity
1	4.32±9.95	5.52±11.1	0.61±0.82
2	4.89±11.9	8.52±24.2	0.58±0.78
3	3.96±9.53	6.24±17.9	0.68±0.86
4	4.10±10.6	4.63±10.6	0.57±0.76
5	3.62±8.55	5.08±11.6	0.61±0.86

Numbers shown for the parameters are MAPEs between the values used to synthesize an image in the validation bed and the estimated values obtained from matching of that image in the testing libraries.

### Estimating Microtubule Parameters for Images of Eleven Cell Lines

**Figure 4:**
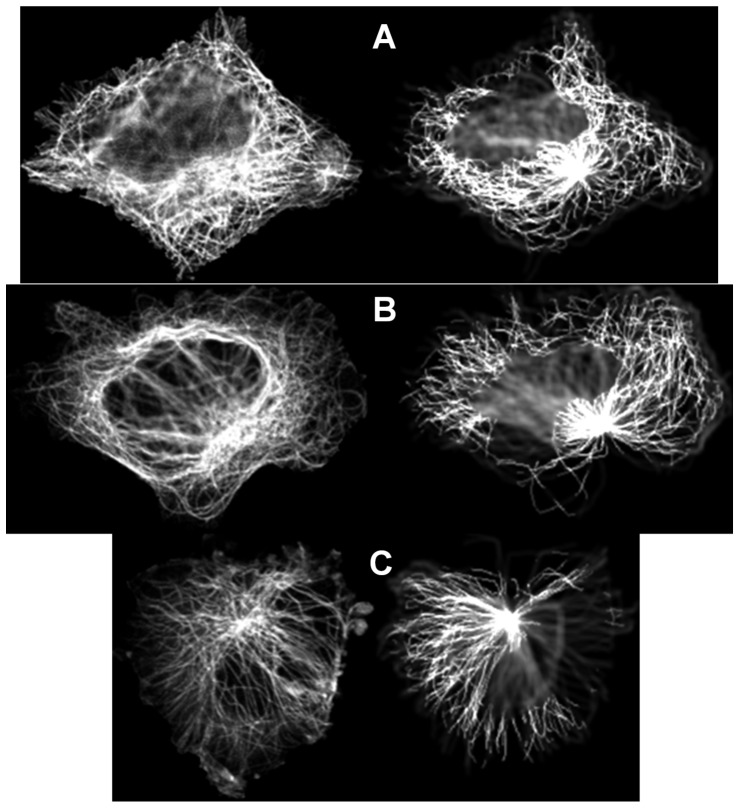
Examples for estimating parameters values by matching to simulated images. 2D real images are shown on the left, and center slices of the best-matching 3D synthetic images are shown on the right. (A) A-431 cell line, Number of microtubules = 250, Mean of length distribution = 30 microns, Collinearity = 0.97000; (B) U-2OS cell line, Number of microtubules = 250, Mean of length distribution = 30 microns, Collinearity = 0.98466; (C) U-251MG cell line, Number of microtubules = 250, Mean of length distribution = 20 microns, Collinearity = 0.99610.

**Figure 5:**
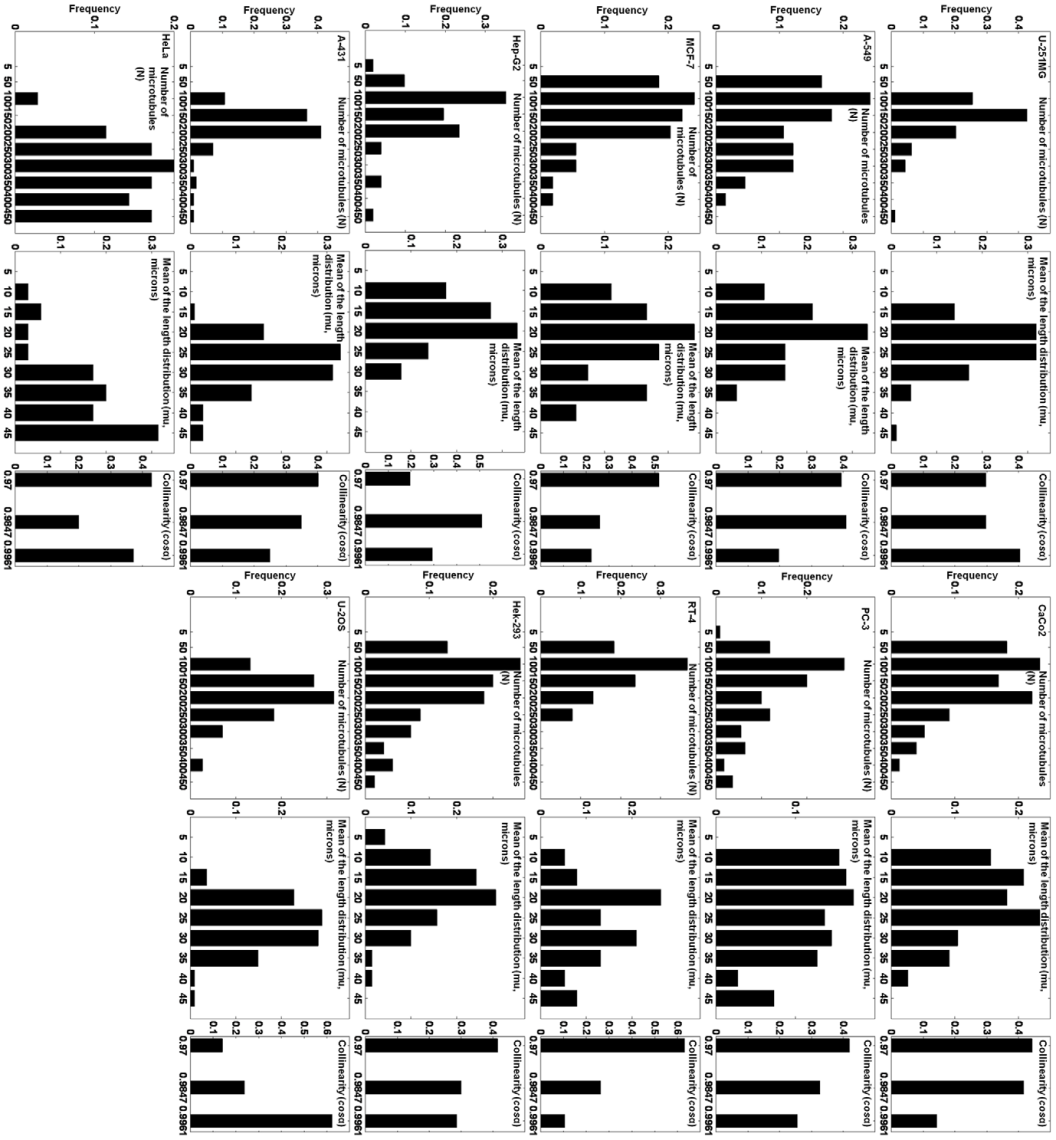
Frequency distributions of all estimated parameters from real 2D images for all cell lines. There are two sets of three columns for the model parameters (number of microtubules, mean of the length distribution and collinearity) in each row. The cell lines (from top to bottom) are U-251MG, A-549, MCF-7, Hep-G2, A-431 and HeLa in the left column, and CaCo2, PC-3, RT-4, Hek-293, and U-20S in the right.

**Figure 6:**
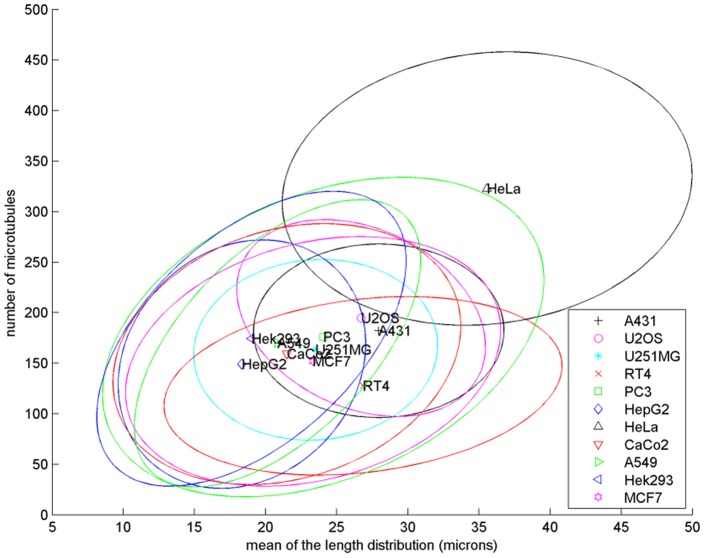
Comparison of the bivariate distributions of the estimated model parameters of the eleven cell lines. The ellipses are centered at the bivariate means of the two parameters and contain about 67% to 80% of the cells for a particular cell line (at most 1.5 standard deviations from the means).

3D microtubule model parameters were estimated from 2D fluorescence microscopy images of eleven cell lines collected as described previously [1], with the application of the whole framework including library generation, feature calculation and matching (see Methods). This dataset consisted of 112 A-431 cells, 114 from U-2OS cells, 94 U-251MG cells, 38 RT-4 cells, 110 PC-3 cells, 51 Hep-G2 cells, 35 HeLa cells, 77 CaCo2 cells, 66 A-549 cells, 70 Hek-293 cells and 54 MCF-7 cells. **Figure 4** shows examples of query images and the corresponding images synthesized using the parameters estimated from them. Note that the synthetic images are not “exactly” the same as the corresponding real ones in every single microtubule, because the goal of the generative models is to learn the underlying distribution of microtubules from which the real images were drawn. Hence from **Figure 4**, we can see that synthetic images are similar to real ones in terms of the distribution of microtubules. There is an underlying assumption that the cells from the same cell line tend to have some level of consistency in the distribution of microtubules. Therefore, we measured the *coefficient of variation* (the ratio of the standard deviation to the mean) for the estimated parameters of real cells. The resulting values for the number of microtubules ranged from 0.28 to 0.60 and from 0.21 to 0.43 for the mean of the length distribution. We show the frequency distribution of each of the three parameters for every cell line in **Figure 5**. It shows that most of the cell lines have quite close to a normal distribution for both the number of microtubules and mean length. Some may deviate a little to have a Gamma distribution-like shape. For the collinearity, due to the computational efficiency, we only used three candidate values in the library of synthetic images, so we cannot draw any significant conclusions. The scatter plot of the two dimensional parameter space (number of microtubules and mean length) estimated from those cell lines is shown in **Figure 6**. The plot shows the variation in number of microtubules, in mean length and in joint correlation of the two. We will compare them in the next section.

### Comparing Microtubule Distributions Across Eleven Cell Lines

#### Comparing bivariate distributions of the number of microtubules and the mean of length

**Figure 7:**
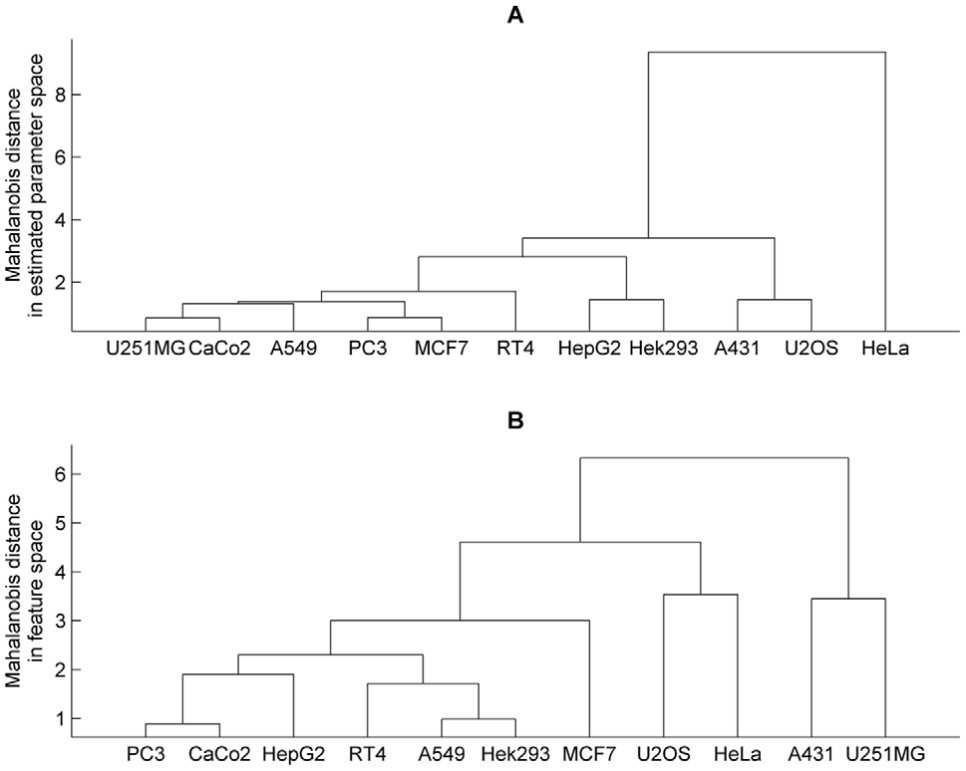
Hierarchical clustering trees of eleven cell lines. The trees were built on the pairwise *Hotelling's T^2^* statistics from (A) the testing of the bivariate distributions of the estimated number of microtubules and mean length and (B) from the testing of the bivariate distributions of the first two principal components of the multivariate features computed from the real images.

We compared the bivariate distribution of the estimated number of microtubules and the mean of length across different cell lines. We first compared the covariances using *Box's M* test. The *p*-value for this comparison was≈0 which indicates that we can readily reject the null hypothesis of homogeneity of covariances. Next, we used the pairwise *Hotelling's T^2^* test to test whether there were significant differences between the bivariate means of the distributions between cell lines. Because there is strong imbalance of the number of cells among different cell lines, we repeated the pairwise testing 100 times each for subsamples of 35 cells (the minimum number of cells for a cell line) for every cell line and then the minimum *p*-values from the repeats (after Bonferroni correction) were reported. All the pairwise *p-*values were then adjusted using family-wise Bonferroni correction for multiple testing [10]. We show the *p*-values in the lower triangular part of Table **3**, and the ones denoted with “*” indicate significant differences. In addition, given the *Hotelling's T^2^* statistics, we built a hierarchical clustering tree shown in **Figure 7 (A)**, and the rows and columns of the lower triangular part of Table **3** are sorted according to the tree.

**Table 3:** Statistical tests of the model parameters and the features between cell lines.

*p*-values	U-251MG	CaCo2	A-549	PC-3	MCF-7	RT-4	Hep-G2	Hek-293	A-431	U-2OS	HeLa
U-251MG (94)	NA	0*	0*	0*	0*	0*	6.1e-13*	1.1e-10*	5.8e-6*	9.8e-10*	0*
CaCo2(77)	1	NA	*1*	*1*	*0.86*	0.045*	6.3e-6*	5.5e-3*	0*	0*	0*
A-549(66)	0.077	1	NA	*1*	0.012*	*0.32*	*0.12*	*1*	0*	7.3e-12*	0*
PC-3(110)	1	1	1	NA	*0.62*	*1*	7.6e-4*	*1*	0*	0*	0*
MCF-7(54)	1	1	1	1	NA	4.9e-5*	9.2e-12*	3.1e-6*	0*	0*	0*
RT-4(38)	0.11	0.030*	5.4e-4*	0.067	1	NA	7.3e-5*	*1*	0*	0.029*	3.1e-5*
Hep-G2(51)	5.7e-4*	1	1	2.0e-3*	0.081	1.0e-4*	NA	0.020*	0*	0*	0*
Hek-293(70)	4.3e-3*	0.92	1	0.26	0.12	2.0e-9*	1	NA	0*	4.1e-7*	0*
A-431(112)	1.5e-4*	8.7e-6*	2.7e-9*	0.012*	0.059	7.1e-3*	0*	0*	NA	7.0e-6*	0*
U-2OS(114)	2.6e-7*	1.1e-5*	1.9e-4*	0.12	4.1e-3*	8.6e-6*	0*	2.9e-11*	1	NA	6.1e-13*
HeLa(35)	0*	0*	0*	0*	0*	0*	0*	0*	0*	0*	NA

The lower triangular part of the table is for the testing of equality of the bivariate mean of the distribution of two estimated microtubule parameters (number of microtubules and mean of length) between cell lines using Hotelling's *T^2^* test. The upper part (Italic) is for testing of the equality of the bivariate mean of the distribution of the first two principal components (learned from and representing the multivariate distribution of features on real cells). The rows and columns of the table are sorted according to the tree from **Figure 7 (A)**. The *p*-values are adjusted according to the family-wise Bonferroni correction for multiple testing. The “*” denotes cell lines which differ at significance level *alpha* = 0.05. The number in the parenthesis of the first column is the number of cells from each cell line.

#### Comparing multivariate distributions of numerical features on real images

As a comparison to these statistical tests of indirect parameter estimates, we repeated the calculations mentioned above using features calculated directly from real cell images. We used the first two principal components, which accounted for 99.99% of the total variance in feature space, to represent the multivariate features. The *p*-value for covariance homogeneity test was≈0. The *p*-values for the pairwise *Hotelling's T^2^* test of bivariate means of distribution of the first two principal components (to represent multivariate means of the distribution of features) are in the upper triangular part of **Table 3**. The hierarchical tree on the basis of the statistics is displayed in **Figure 7 (B)**, but the rows and columns of the upper triangular part of Table **3** are also sorted according to the tree in **Figure 7 (A)** for consistency with the lower triangular part. The comparison using image features indicates that 44 out of 55 show statistically significant differences (of which 27 were comparisons involving HeLa, A-431 and U-2OS). However, when the estimated model parameters were compared (in the lower triangular part of Table **3** and **Figure 7 (A)**), 31 out of 55 comparisons showed statistical significance. Of these, 24 were comparisons involving HeLa, A-431 and U-2OS cells. Thus when these cells are subtracted (since they are clearly different from the rest of the cell lines), the number of presumed differences dropped from 31 to only 7. We believe that this is an indication of the utility of the method: the full set of features reflects a variety of differences among the cell lines in a range of possible (latent) parameters not necessarily directly relevant to microtubule distributions (such as cell size and shape and nuclear size and shape). The model parameter estimation is, on the other hand, able to ignore these, and focuses on microtubules. In that case, eight of the cell lines appear to be fairly similar. Consideration of all of **Table 3**, **Figure 7 (A)** **and** **Figure 6** suggests that HeLa, A-431 and U-2OS are very different from those eight but A-431 and U-2OS are close to each other in the estimated model parameter space. The differences among the three groups can largely be accounted for by differences in total polymerized tubulin from **Figure 6**. Similarly, among the group of eight, we can observe that RT-4 appears to have fewer, longer microtubules, Hep-G2 appears to have lower total tubulin, and Hek-293 appears to have shorter microtubules.

#### Correlation between the estimated amount of polymerized tubulin and total tubulin fluorescence

**Figure 8:**
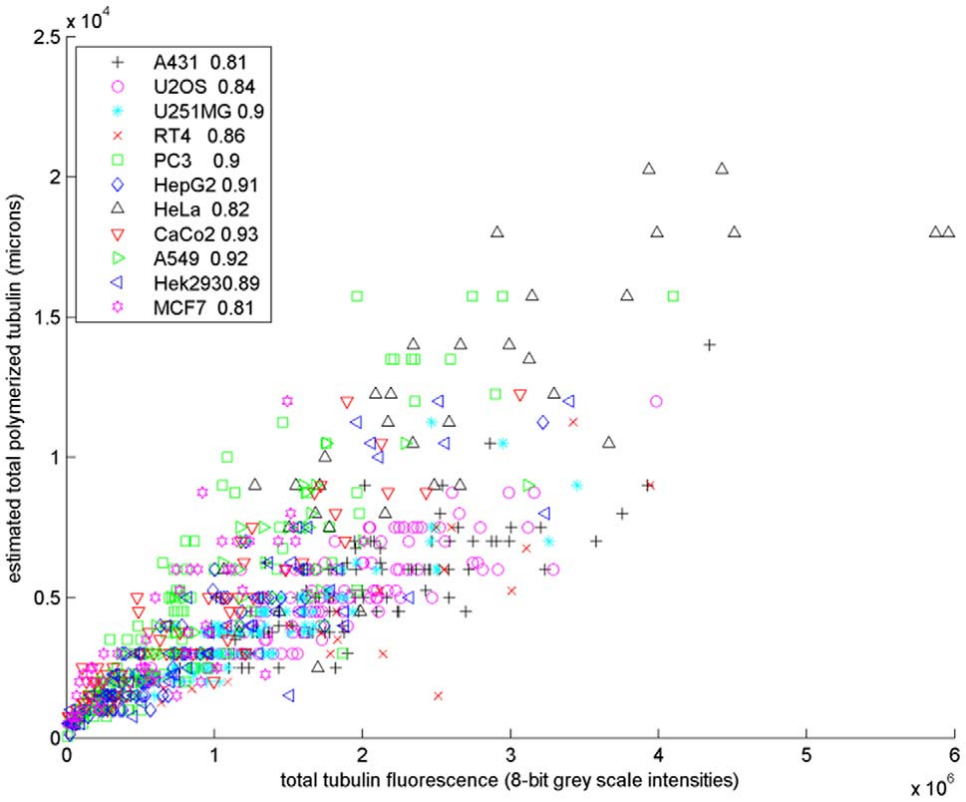
Scatter plot of the estimated total amount of polymerized tubulin (the product of the estimate number of microtubules and the mean length) versus the total tubulin fluorescence intensity of real images from eleven cell lines. The correlation coefficient for each cell line is shown in the legend.

We compared the amount of polymerized tubulin, estimated as the product of the number and mean length of the microtubules, to the total intensity of each cell image. The plot of these two quantities for real cells from eleven cell lines is shown in **Figure 8**. The high correlations demonstrate the consistency between the estimated and real amount of polymerized tubulin and the effectiveness of our methods.

## Discussion

We have developed an automated method to estimate 3D microtubule model parameters from 2D confocal immunofluorescence microscopy images in an indirect manner. The method is dependent on the 3D structure of the cell and the nucleus, and the centrosome location. We describe an automated approach in the method to generate an approximate 3D cell and nuclear morphology using only the 2D microtubule image and 2D nucleus image acquired at the center (half height) of the cell. We applied this method to generate distributions of microtubules in cells and utilized an indirect feature matching algorithm to estimate model parameters from 821 images of cells and 11 cell lines. Then the two quantitative parameters, number of microtubules and mean length of microtubules, were compared across cell lines. These two parameters are important because they demonstrate the fundamental physical characteristics of microtubules in cells.

To our knowledge, this study is the first attempt to quantify the number and mean of the length distribution of microtubules in intact cells across different cell lines. Methods such as electron microscopy can image intact cells, but have interference from other cell components [11]. More invasive methods of preparation such as extraction of the microtubule network can allow electron microscopy to generate traceable images, but are no longer representative of intact cells [12]. Fluorescence microscopy, on the other hand, can be used to obtain information about proteins at monomer-level resolution of localization without interference from other cell components in intact cells with high-throughput data.

One reason for studying microtubule distributions across cell lines is to begin to search for explanations of how expression of microtubule-associated proteins (MAPs) may account for any differences observed. The expression levels of many proteins vary across cell lines [13], and there are cell-specific proteins that regulate microtubules [14], [15]. In this paper, the cell lines chosen are from varying lineages, such as mesenchymal, epithelial and glial tumors, which may differ in their expression of MAPs. Our analyses show that some cell lines do have significant differences in the estimated parameters of the number and length distribution of microtubules. In future work, we hope to establish whether and how these differences results from variation in expression of specific MAPS.

**Figure 9:**
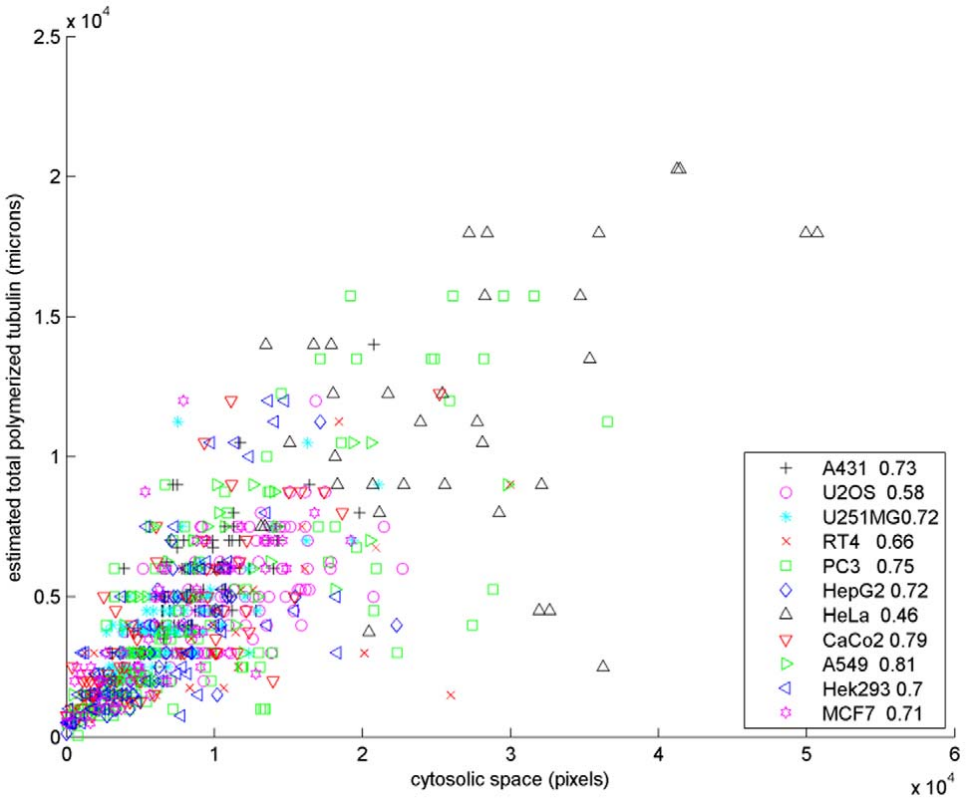
Scatter plot of the estimated total amount of polymerized tubulin versus the area of cytosolic space (sum of pixels) for real cells from eleven cell lines. The correlation coefficient for each cell line is shown in the legend.

There is evidence that the number and length of microtubules are correlated with the size of the cell [16], [17]. We therefore computed the area of the center slice (sum of pixels of the binary image) as the value reflecting the size of cytosolic space of the cell, for each of the cell lines. To quantify the correlation, we computed the correlation coefficient between the estimated total polymerized tubulin and the area of cytosolic space for each cell line. The plot of these two quantities for all cells is shown in **Figure 9**. The correlation coefficients varied from 0.46 to 0.81 which are intermediate to high. They add more confidence to the estimates of our automated approach and further confirm the existing hypothesis using alternative approaches.

The methods described here have potential applications in a range of experimental approaches. For example, microtubule interacting drugs (mitotic inhibitors) are commonly used for cancer chemotherapy, and our method could provide a quantitative measure of the effects of these drugs on different cancer cell types. It also could be used in high-content screening to distinguish different types of effects of compounds that disrupt microtubule dynamics.

Finally, we note that our estimation procedure is only appropriate for images and cell lines for which the majority of microtubules originate at the centrosome because we explicitly modeled all microtubules as starting from it. Therefore, the centrosomes may appear more focused in some synthetic images compared to the corresponding experimental ones for cell types that are less organized by centrosomes. Future work could include modifications to our modeling procedure so that it can be used with a more diverse set of experimental images and cell lines.

## Materials and Methods

### Data Acquisition

#### 3D image data of HeLa cells

We used 3D images of HeLa cells previously obtained by three color confocal immunofluorescence microscopy to visualize three cell components: the cell membrane, nucleus and microtubules [18]. The original pixel size was 0.05 microns, and the images were downsampled for computational efficiency to 0.2 microns.

#### 2D images of eleven cell lines

The data used here are confocal immunofluorescence microscopy images of fixed and interphase cells of eleven different cell lines: A-431, U-2OS, U-251MG, RT-4, PC-3, Hep-G2, HeLa, CaCo2, A-549, Hek-293 and MCF-7 from the HPA. They are human cell lines widely used in current research. The images were acquired as described previously [1], and the cell lines were obtained from ATCC-LGC Promochem (Boras, Sweden) except that the first two were obtained as described previously [1]. The images are analyzed as 8-bit TIFF images, with two files each obtained using a different emission wavelength of fluorescence from a single image field. These two channel files show the cellular probes/organelles used as references: (i) anti-tubulin antibody as internal control and marker of microtubules and (ii) DAPI for the nucleus. Each of the field images is of size 1728×1728 for the first three cell lines and 2048×2048 for the rest of eight, and the pixel size is 0.08 microns in the sample plane. The field images were then also downsampled for computational efficiency to 0.2 microns.

### Computational Methods

#### Cell segmentation for cell size calculation and 3D morphology generation

The field images were segmented into single cell regions using a seeded watershed method on the tubulin channel with the nuclei in the nuclear channel as seeds. The 2D cell and nuclear boundaries were found by thresholding the single cell regions and the nuclei respectively. These were used for cell size calculation and for 3D morphology generation (see below).

#### Point Spread Function (PSF) estimation

The confocal PSF was generated computationally based on a theoretical model using the SVI PSF calculator for the Zeiss LSM 510 confocal microscope for the first three cell lines and the Leica SP5 for the other eight cell lines (http://www.svi.nl/NyquistCalculator). The pinhole size was set to 1 Airy Unit for the Zeiss and 285.16 nm for the Leica. The numerical aperture was 1.4 and the emission-excitation data used to generate the PSF was for the Alexa555 dye (http://probes.invitrogen.com/handbook/boxes/0442.html). The PSF is used to convolve on the generated raw image of distribution of microtubules to account for the digital blurring from microscopy imaging.

#### Centrosome location detection

The 3D coordinate of the centrosome was estimated by breaking the problem into two parts. First, the XY-coordinate was estimated and then the Z-coordinate. The XY-coordinate was chosen as the pixel with the maximum intensity value in the vicinity of the nucleus after smoothing with an averaging filter of size 25 pixels on the tubulin channel image (as for cell image). For the Z-coordinate, we used linear regression to estimate the location as a function of the following predictor variables: (i) Maximum intensity of the microtubule image, (ii) Mean intensity of the microtubule image, and (iii) pixel intensity of the XY coordinate in the microtubule image. The coefficients of the linear regression were estimated from the 3D HeLa images where the 3D centrosome as described previously [8]. The estimated centrosome is then used to act as an organizer for microtubules and all generated microtubules start from it.

#### Estimation of single microtubule intensity

The single microtubule intensity for each cell line was estimated using the method described previously [9]. It is then used to scale the intensity of synthetic image up to that of the real image.

#### 3D cell and nuclear morphology generation

In order to estimate the cell shape, we firstly required the following two estimates: (1) the cell shape at the bottom, where the cell membrane interacts with a substrate (e.g. petri-dish), and (2) cell shape decay from the bottom of the cell to the top.

For estimating the bottom shape of the cell, we used the microtubule channel image acquired at the center of the cell, i.e. z = Z/2, where Z is the height of the cell in pixel dimensions. This image contains information about the cell boundary at the bottom-most region because the out-of-focus light from the bottom slice is visible in the center slice (as microtubules being of relatively lower intensity). Hence, the boundary of the bottom slice (bottom shape) was found by thresholding for above zero intensity pixels. (see **Figure 3 (A)** for an example). Next, we represented the cell shape decay by estimating cell shape pixel area as a function of height of the cell, i.e. A(z). This function was estimated from the average area profile of the 2D slices in the 3D HeLa stack (data not shown) to be A(z) = 2^−z^*Area, where *Area* is the pixel area of the bottom slice, and *z* is the distance from the bottom. Since the cell tapers from the bottom shape to the top (because of the presence of a nucleus), we modeled the 3D cell shape by interpolating from the bottom shape of the cell to a smaller ellipse inside the cell whose major axis was aligned with that of the cell. This interpolation was done using distance transform based shape interpolation [19]. Given the height of the cell and the z-sampling step-size (0.2 microns, 1 pixel volume per stack), we discretized this model at varying z by choosing interpolated shapes that have areas that match the estimated area profile A(z) from the 3D HeLa stack. **Figure 3 (C)** shows an example of generated 3D cell shape containing 8 stacks (height of 1.6 microns). The 3D nuclear morphology was generated based on the same procedure above using the nucleus channel image (**Figure 3 (D)**). Then microtubules are generated conditioned on the approximate 3D cell and nuclear shape.

#### Growth model of microtubule patterns

The growth model of microtubule patterns (**Figure 1**) is similar to the one described previously [8], with three modifications: (i) the Erlang distribution was used for microtubule lengths since, unlike the Gaussian distribution, it has only one free parameter; (ii) if the microtubule is required to make a turn in 3D space such that the 3D angle is greater than 63.9 degrees with *cosine* value of 0.44 (this value is chosen manually to account for appearance of real microtubules as well as the generability of the model), the growth procedure for it is terminated; and (iii) if within a consecutive 30 steps (about 6 microns) of growth of a microtubule, there are more than 3 pairwise vector angles that are greater than 120 degrees, the growth procedure for it is terminated. In order to ensure that the input parameters are exactly the same as the output parameters, we use the following algorithm to generate the images.

1. **Input parameters:** number of microtubules (n), mean of the length distribution (mu), collinearity (α);
2. Sample *n* lengths from Erlang distribution;
3. Sort lengths from longest to shortest;
4. Iterate until all lengths are generated, starting with the longest microtubule:

**for** *i* = 1 to *n* **do**

**if** storage has microtubule of desired length generated then

  use the generated microtubule length;

  remove chosen microtubule from storage;

**continue**, to the next microtubule.

**end if**

**loop**

  Generate a microtubule using the method in **Figure 1**.

**if** the desired microtubule length cannot be generated then

   add to storage and re-generate the microtubule.

**if** repeating 100 times still does not generate a microtubule of desired length then

**return** declare “input parameters cannot be generated”.

**end if**

**end if**

**end loop**

**end for**

Finally the generated image was convolved with the estimated PSF and was then multiplied with the corresponding estimated single microtubule intensity to make the intensity comparable to real images.

#### Library generation

As described previously [8], a library of synthetic images was generated for each cell geometry (cell shape and nucleus shape) and contained all combinations of the parameter values below (resulting in a total of 810 synthetic images). The values were chosen by experience to account for the appearance of real microtubules as well as the generability and computational efficiency of the model):

- Number of microtubules = 5, 50, 100, 150, 200, 250, 300, 350, 400, 450;
- Mean of length distribution = 5, 10, 15, 20, 25, 30, 35, 40, 45 microns;
- Collinearity (*cosα*) = 0.97000, 0.98466, 0.99610;
- Cell Height = 1.2, 1.4, 1.6 microns.

#### Features and matching

For each 2D real cell image and all the central 2D slices from its 3D simulated images in the library, 2D versions of the features that were used previously [8] were calculated. Detailed information about the implementations of the 2D version of the features have been presented [20]. In addition, we appended the feature set with edge features, which were some histogram features calculated on the gradient magnitude and gradient's direction after convolving each 2D image with Prewitt operator. Following the feature computation, we calculated the normalized Euclidean distances between the feature vector of the real image and those of its simulated images for matching. The set of parameters that was used to generate the simulated image with the minimum distance was used as estimates of the parameters of distribution of microtubules in that real image [8].
